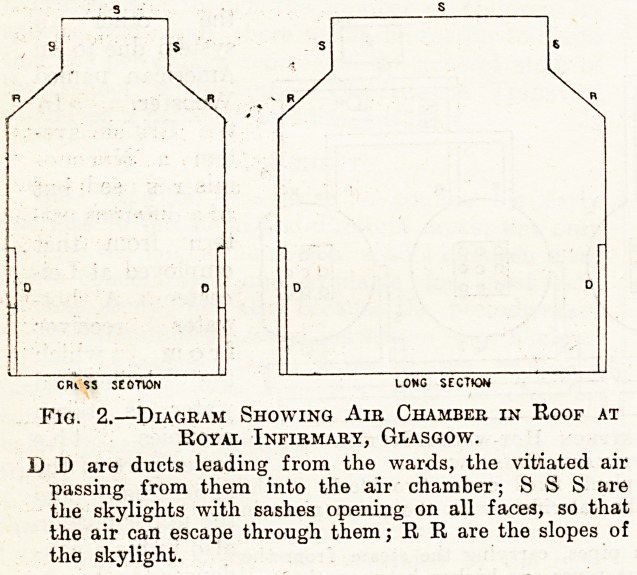# The Royal Infirmary, Glasgow

**Published:** 1915-11-13

**Authors:** 


### THE HEATING OF HOSPITALS.*

[NOTE.—In a series of articles the writer proposes to describe the different systems of heating that are employed in hospitals. He will be pleased to answer any questions through "The Hospital," bearing upon the subject of heating or ventilation. Each system will be illustrated and described as exemplified at some hospital where it has been applied.

### V.—The Royal Infirmary, Glasgow.

### HOT-WATER RADIATORS ON THE THERMO-SYPHON SYSTEM AND LOW-PRESSURE STEAM-RADIATORS.

In the Royal Infirmary at Glasgow, the north and centre blocks are heated by hot-water radiators, the south block by low-pressure steam. There are three Lancashire boilers, each 30 ft. long by 8 ft. 6 in. in diameter, working at a pressure of 125 lbs. per square inch. All the steam is used for heating purposes, none for driving engines or for power in any form. The pressure of the steam is reduced in the boiler-room, by one of the usual reducing-valves, to 60 lbs. per square inch. For the hot-water radiators there are three calorifiers in the basement under the north block, and two under the centre block. Steam is led to the calorifiers at 60-lb. pressure, and is reduced to 25 lb. by reducing-valves before entering them. The heating-system in each block is separate and distinct. From each set of calorifiers a hot-water main is carried to the top of the building, where it is connected to a ring-main and the usual expansion-tank. A second ring-main runs round the block in the basement and is connected to the return-main leading to the battery of calorifiers. The radiators are connected, in various ways, according to their position, between the two ring-mains, and water flows continually through the whole system when the calorifiers are at work; the motive-power being the difference in weight between the column of water in the hot-water pipe and that in the radiators and return-pipe. Fig. 1 shows diagramatically the arrangement of the two batteries of calorifiers, the flow and return mains, the steam-supply pipes, the radiators, and the expansion-tanks.

Fig. 1.—Diagram showing the Thermo-syphon Hot-water System used in Part of the Royal Infirmary, Glasgow.

The diagram shows two sets of heating plant, fixed under two blocks of the infirmary. The circles are sections of the calorifiers with the steam pipes in the centre.

S S S S are the main and branch steam pipes, carrying the steam from the boilers at the calorifiers; the main steam pipes are broken between the two sets of calorifiers to show that the two blocks are some distance apart. C C C are the calorifiers. D D D the drain pipes carrying away the condensed steam. F F F are the hot-water flow pipes. R R R the return pipes from the radiators. H H H are the radiators. X X are the expansion tanks.

The principle is the same as that employed in the Reck system; the advantage claimed for the latter being the smaller pipes that can be employed owing to the greater velocity at which the water is made to flow by the Reck motive-pipe. Higher velocity of flow of water could be obtained in the thermo-syphon system by increasing the temperature at which the hottest water issues from the calorifier; but unless some arrangement was made similar to that with the Reck system in the Glasgow hospitals, to regulate the temperature of the water in the radiators, there would be the same troubles as with high-pressure steam-heated radiators, as well as difficulties in other ways. The great feature of the thermo-syphon system is its simplicity. There are two separate low-pressure steam systems in use in the hospital; one similar to that at Leicester, and the other a system due to an American named Webster. In the British system a Nuconomiser is used, but of a different pattern from that employed at Leicester. A hot-water receiver from which the boiler-feed water is taken occupies the lower part of the apparatus, and the upper portion also forms a condenser for the exhaust steam, as in the arrangement at Leicester. The internal construction of the apparatus is, however, different. In place of the U tubes there is a large perforated pipe through which the exhaust steam passes. The large pipe is surrounded and supported by perforated plates. The exhaust steam enters at the top, the cooling-water also enters at the top, both pass downwards together, the steam being condensed, and the hot water which the steam and cooling-water become, passing into the receiver below. As at Leicester, there is an automatic valve controlling the flow of cold water into the apparatus, the valve being worked by a float in the hot-water receiver.

There is an auxiliary tank, from which the water which is employed for condensing the steam in the Nuconomiser is led, forced by a very ingenious automatic pump. The Nuconomiser and accessories form a central heating-station as at Leicester, though there is only one Nuconomiser. There is

* Previous articles appeared on July 31, September 11, and October 2 and 16, 1915.

one large pipe carrying the condensed water and steam from the calorifiers, steam traps, etc., down to the heating-station, where vacuum pumps are employed as at Leicester. A smaller pipe rises from this main some distance above the heating-station, and from this steam is taken to heat the nurses' home in winter, reinforced by live steam from the boiler reduced to 4½-lb. pressure when required. When heat is not required in the nurses' home the exhaust steam or vapour passes to the Nuconomiser. The vapour also, with live steam at reduced pressure from the boiler, provides the laundry with hot water through a feed water-heater. There is a Green's "Economiser" fixed in connection with the boiler, which is employed to heat the boiler-feed water when the exhaust vapour is required for the nurses' home. The arrangement of the radiators is very similar to that at Leicester, but the flow of steam is controlled by thermostats

Fig. 2.—Diagram Showing Air Chamber in Roof at Royal Infirmary, Glasgow.

D D are ducts leading from the wards, the vitiated air passing from them into the air chamber; S S S are the skylights with sashes opening on all faces, so that the air can escape through them; R R are the slopes of the skylight.

in each ward. The thermostat does the same automatically as the switch-valve described in the Leicester article. The flow of steam to each radiator is controlled by two valves, one regulated by hand in the usual way, and the other automatically by the thermostat. The automatic valve is a plunger which is moved by a spring and by the difference between atmospheric pressure and that produced by the vacuum pumps. When the system is working properly, and the pumps are producing the required vacuum, and when also the thermostat in the ward moves so as to close the connection with the vacuum-pump, the difference between the pressure of the atmosphere and that produced by the vacuum-pump overcomes the tension of the spring, opens the valve, and allows steam to pass into the radiator. If the temperature in the ward rises to the figure at which the thermostat is set, the thermostat makes connection between the vacuum side of the plunger and the atmosphere, breaking the vacuum, the valve then being closed. The thermostat is similar to that at Leicester.

The writer understands that in Glasgow the thermostats do not act well unless each one is frequently cleaned. The atmosphere of Glasgow contains a good deal of matter in suspension, some of which gets into the thermostats and prevents the rods which operate them from moving. One thermostat controls all the radiators in each ward.

### Ventilation.

The arrangement for ventilating is interesting. At the top of the building there is a large air-chamber, shown in cross-section and longitudinal section in fig. 2, with skylights and windows occupying a large portion of the roof, very much on the lines of skylights on board ship. The skylights and windows can be opened individually. Air-ducts from all the wards lead the vitiated air into the air-chamber, from which it escapes through the windows and skylights. It is found by opening the windows and skylights on either side, according to the direction of the wind, that all the ventilation required is obtained. A certain amount of experience will be necessary to arrange the skylights so as to obtain the sucking action required. If the wind at the time is allowed to blow into the air-chamber, the vitiated air will be forced down the ducts into the wards, instead of being sucked out. By arranging, however, that the wind blows past an open skylight, air will be caused to flow out through the skylight, the vitiated air coming with it.

## Figures and Tables

**Fig. 1. f1:**
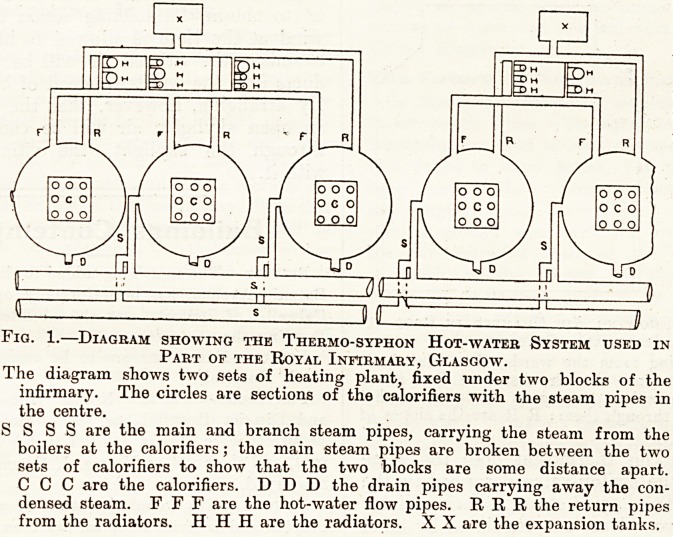


**Fig. 2. f2:**